# The association between health literacy and Health Information-Seeking Behavior among pulmonary nodule patients: a serial mediation of illness perception and self-efficacy

**DOI:** 10.3389/fpubh.2026.1773518

**Published:** 2026-03-18

**Authors:** Jinting Sun, Qian Zhao, Yanxia Han, Chang Zhu, Hongying Qian, Rui Liu, Siying Zhou

**Affiliations:** 1 Department of General Surgery, The First Affiliated Hospital of Soochow University, Suzhou, China; 2 Department of Pulmonology, The First Affiliated Hospital of Soochow University, Suzhou, China; 3 Department of Nursing, The First Affiliated Hospital of Soochow University, Suzhou, China

**Keywords:** health literacy, illness perception, information-seeking behavior, pulmonary nodules, self-efficacy, structural equation modeling

## Abstract

**Objective:**

This study aimed to examine the association between health literacy and Health Information-Seeking Behavior (HISB) among patients with pulmonary nodules (PNs). It further assessed whether illness perception and self-efficacy were associated with this relationship using a theoretically specified serial mediation model informed by the Information-Motivation-Behavioral Skills (IMB) framework.

**Methods:**

This cross-sectional study was conducted from February to June 2024. Patients with PNs were recruited from two tertiary hospitals in Suzhou, China, using convenience sampling. Structural equation modeling (SEM) was applied to test hypothesized associations among variables. Bias-corrected bootstrapping was used to estimate direct and indirect effects, including serial indirect effects consistent with the hypothesized ordering. Multi-group analysis examined whether model estimates differed by educational level. Reporting followed the STROBE guidelines.

**Results:**

Overall, 321 patients completed the survey. The mean score of HISB was 131.85 (SD = 34.96). HISB showed modest positive correlations with health literacy (*r* = 0.464, *p* < 0.01) and self-efficacy (*r* = 0.497, *p* < 0.01), and negative correlation with illness perception (*r* = −0.429, *p* < 0.01). The SEM showed excellent fit (*χ*^2^/df = 1.46, RMSEA = 0.038, CFI = 0.982). Health literacy showed association with HISB (*β* = 0.477, *p* < 0.001). Indirect associations were observed via self-efficacy [*β* = 0.110, 95% CI (0.062, 0.173)] and illness perception [*β* = 0.065, 95% CI (0.035, 0.108)]. A statistically significant but modest serial indirect effect was observed [*β* = 0.021, 95% CI (0.009, 0.040)], consistent with the hypothesized model. Multi-group analysis supported configural invariance across education levels, although the strength of some associations varied.

**Conclusion:**

This study found both direct and indirect associations between health literacy and HISB among patients with PNs. The findings suggest that interventions that providing literacy-sensitive support, address maladaptive illness perception, and strengthen self-efficacy may help foster adaptive information-seeking and improve long-term surveillance adherence and psychological outcomes.

## Introduction

With the widespread adoption of computed tomography (CT) in routine clinical practice and lung cancer screening programs, pulmonary nodules (PNs) are detected at high rates worldwide, with reported detection rates ranging from 30 to 59% (1, 2). Although most nodules ultimately prove benign, a subset may represent early-stage lung cancer, necessitating prolonged surveillance through repeated imaging over months to years (3, 4). Patients frequently experience psychological distress; common reactions include anxiety, decisional conflict, and difficulty interpreting risk information (5–7). Such uncertainty can not only affect emotional well-being but also have a substantial impact on long-term health outcomes (8). Accordingly, while some patients actively seek information to reduce uncertainty, others may avoid health-related information as a coping response to distress. Understanding how patients seek, process, and use health information during surveillance is therefore critical for adherence to follow-up protocols, emotional well-being, and overall health management.

Health Information-Seeking Behavior (HISB) serves as an important coping strategy for managing uncertainty in patients with PNs. HISB is defined as the active process through which individuals locate, appraise, and apply health-related information to comprehend their condition, guide decision-making better, and manage associated risks (9). In an era where information is readily accessible, actively HISB is a key adaptive strategy for navigating uncertainty and understanding complex medical conditions (10, 11). For patients with PNs, who often experience heightened anxiety and limited understanding, HISB can alleviate distress and foster a sense of control (6, 12). By bridging knowledge deficits and clarifying management options, this process facilitates the transition from passive receipt of care to active participation in long-term self-management (13).

Nevertheless, the efficacy of HISB is fundamentally shaped by health literacy. Health literacy encompasses the capacity to obtain, process, comprehend, and apply health information for informed decision-making (14). Higher health literacy has consistently been associated with improved outcomes across diverse chronic illness populations (15). However, a meta-analysis revealed only a modest correlation between health literacy and HISB (16), suggesting that additional psychological and motivational mediators warrant exploration.

Illness perception, which encompasses cognitive and emotional representations of a health threat (17), additionally exerts considerable influence on the propensity to engage in HISB (18). These cognitive and emotional representations influence how individuals assess health threats and respond through coping behaviors, including HISB (19). Patients with PNs often receive ambiguous diagnostic information. For these individuals, threatening illness perception, such as high uncertainty and a perceived lack of control, may reduce their willingness to engage with health information (20). Emerging evidence suggests that health literacy may be associated with less threatening illness perception, which may in turn support more proactive HISB (21).

Furthermore, self-efficacy, defined as confidence in one’s ability to successfully execute specific behaviors (22), constitutes a critical behavioral skills component. Wu et al. proposed that self-efficacy acts as a key mediator linking health literacy and social support to actual health behavior (23). Patients with high self-efficacy are more likely to persist in seeking answers despite complex medical terminology or ambiguous diagnostic results, making self-efficacy a key component in facilitating HISB (24). In addition, by promoting a more coherent and less catastrophic understanding of PNs, health literacy may strengthen patients’ confidence in their ability to engage with health information, thereby enhancing self-efficacy for HISB.

Health literacy, illness perception, and self-efficacy each contributes to the understanding of HISB. However, a comprehensive understanding requires a unifying framework that delineates these synergistic roles. The Information-Motivation-Behavioral Skills (IMB) model provides a parsimonious framework in which health behavior is shaped by information, motivation, and behavioral skills (25). In the context of PNs, health literacy serves as the information component that enables patients to understand their condition. Self-efficacy, embodying the behavioral skills component, determines confidence in executing information-seeking behavior. While the IMB model has been successfully applied to various health behaviors (26, 27), its utility in explaining HISB among patients with PNs remains unexplored.

Although HISB is vital for chronic disease management, research specifically focusing on patients with PNs remains scarce. Furthermore, the pathways through which health literacy, illness perception, and self-efficacy interact to influence HISB within the IMB framework are not well understood in this population. Guided by the IMB model, this study aimed to investigate how health literacy, illness perception, and self-efficacy jointly shape HISB among patients with PNs and to examine the mediation and sequential mediation roles of illness perception and self-efficacy in the relationship between health literacy and HISB.

## Research hypotheses

Grounded in the IMB model and extant literature, it was hypothesized that health literacy would exert both direct and indirect effects on HISB through illness perception and self-efficacy, with the following specific mediation pathways proposed:

*Hypothesis 1*: Illness perception mediates the relationship between health literacy and HISB.

*Hypothesis 2*: Self-efficacy mediates the relationship between health literacy and HISB.

*Hypothesis 3*: Health literacy is associated with HISB through a serial indirect effect via illness perception and self-efficacy.

**Figure 1:**
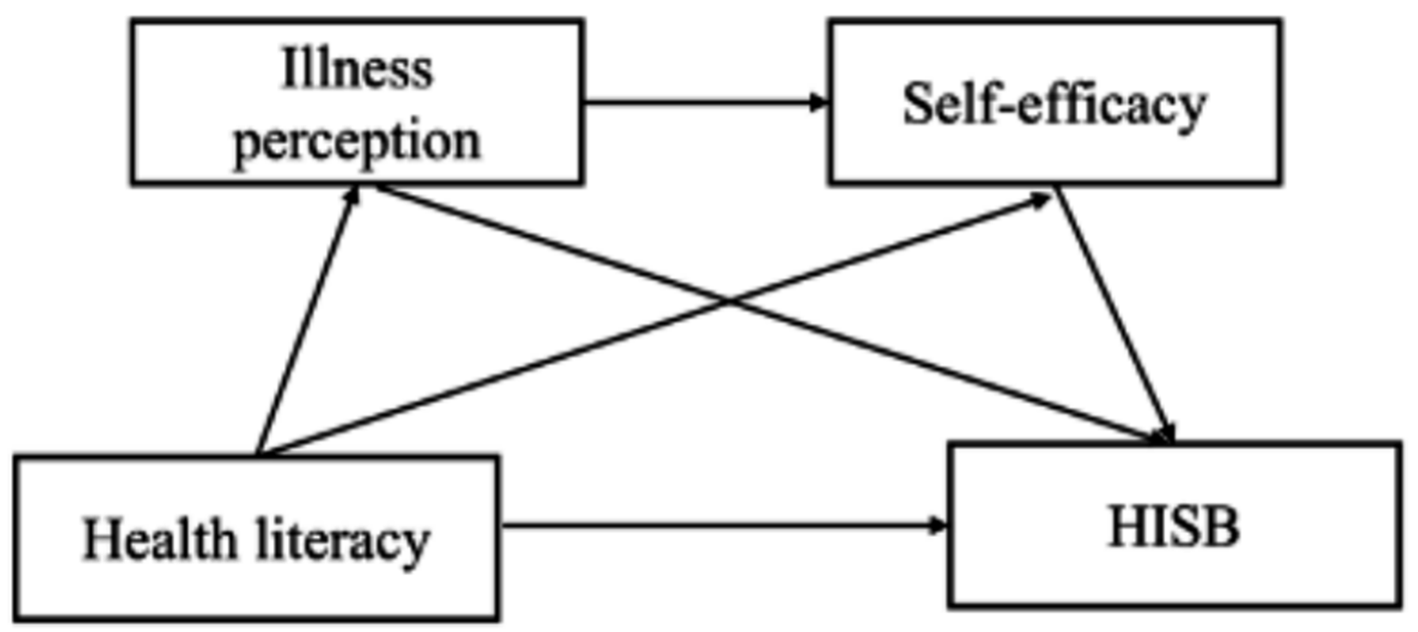
Hypothesized conceptual framework based on the IMB model.

The hypothetical conceptual framework is presented in Figure 1.

## Methods

### Design, setting, and participants

This cross-sectional study was conducted between February and June 2024 in the outpatient clinics of thoracic surgery and respiratory medicine at two tertiary general hospitals in Suzhou, Jiangsu Province, China. A convenience sampling strategy was employed. The inclusion criteria: (1) age ≥18 years; (2) radiologically confirmed pulmonary nodules; (3) ability to communicate in Chinese; and (4) provision of informed consent. The exclusion criteria: (1) established diagnosis of lung cancer; (2) severe cognitive impairment or primary psychiatric disorders; and (3) physical conditions impeding independent completion of the questionnaire. The study protocol complied with the Strengthening the Reporting of Observational Studies in Epidemiology (STROBE) guidelines.

Sample size was calculated using G*Power 3.1 for multiple linear regression (3 predictors, f^2^ = 0.15, *α* = 0.05, power = 0.95), yielding a minimum of 119 participants. To accommodate a 10% invalid questionnaire rate and to meet recommended thresholds for structural equation modeling (≥200 cases; 46), we aimed to recruit 250–300 patients. After excluding cases that did not meet the eligibility criteria or contained substantial missing or inconsistent data, 352 patients with pulmonary nodules were initially enrolled. Of these, 321 valid questionnaires were retained, yielding an effective response rate of 91.2%.

### Measurements

#### Demographic and clinical characteristics

A structured form was used to obtain sociodemographic and clinical characteristics, including age, sex, marital status, educational level, occupation, monthly income, place of residence, nodule type, and duration of disease. Clinical information was verified through medical records.

#### Health Information-Seeking Behavior Scale (HISB)

The Health Information-Seeking Behavior Scale (HISB), originally developed by Zamani et al. (28) and subsequently adapted into Chinese by Sun et al. (29), was used to assess HISB. The 43-item instrument comprises four domains: information needs (14 items), information sources (15 items), attitudes towards health information seeking (6 items), and barriers to information access (8 items). Each item is rated on a 5-point Likert scale(1 = strongly disagree, 5 = strongly agree). Items 1–35 are positively keyed, and items 36–43 are reverse-scored. The total score is calculated as the mean of all item scores after reverse scoring, with higher scores indicating more active HISB. In this study, the scale exhibited excellent internal consistency, with a Cronbach’s *α* of 0.964.

#### Health Literacy Management Scale (HeLMS)

Health literacy was assessed using the Health Literacy Management Scale (HeLMS), initially developed by Jordan et al. (30) and adapted into Chinese by Sun et al. (31). The 24-item scale consists of four domains: information acquisition, communication and interaction, motivation to improve health, and willingness to seek financial support. Items are rated on a 5-point Likert scale ranging from 1 (“unable to do”) to 5 (“without any difficulty”). The total score is calculated as the sum of all item responses (range 24–120), with higher scores indicating better health literacy management capacity. HeLMS was used to assess individual-level health literacy; health-system and organizational determinants were not measured in this study. In this study, the scale exhibited excellent internal consistency, with a Cronbach’s *α* of 0.962.

#### Brief Illness Perception Questionnaire (B-IPQ)

Illness perception was assessed using the Brief Illness Perception Questionnaire (B-IPQ), developed by Broadbent et al. (32) and translated into Chinese by Mei et al. (33). The scale comprises nine items; the first eight are rated on an 11-point Likert scale (0–10). A total score (range 0–80) is obtained by summing these eight items after reverse-scoring items 3 (personal control), 4 (treatment control), and 7 (illness comprehensibility). Higher scores indicate more threatening illness perception. The ninth item is open-ended and is not included in the total score. In this study, the scale exhibited excellent internal consistency, with a Cronbach’s *α* of 0.926.

#### General Self-Efficacy Scale (GSES)

Self-efficacy was measured using the 10-item General Self-Efficacy Scale (GSES), initially developed by Schwarzer et al. (34) and validated in Chinese by Wang et al. (35). Each item is rated on a 4-point Likert scale ranging from 1 (“completely incorrect”) to 4 (“completely correct”). The total score is calculated as the sum of all item scores, with higher scores indicating greater self-efficacy. In this study, the scale exhibited excellent internal consistency, with a Cronbach’s *α* of 0.930.

#### Data collection

A pilot test with 20 pulmonary-nodule patients refined wording and layout; only minor linguistic adjustments were required. During the main data collection phase, trained research nurses sequentially approached eligible outpatients, explained the study purpose and procedures, obtained written informed consent, and administered paper-based questionnaires in a quiet area of the clinic. For participants with reading difficulties, items were read aloud without suggestion. Completed forms were checked on-site for completeness, double-entered into REDCap, and cross-verified to minimise entry error.

#### Ethical approval

This study was approved by the Ethics Committee of the First Affiliated Hospital of Soochow University (Approval No. 2024122). Written informed consent was obtained from all participants after a full explanation of the aims, procedures, and voluntary withdrawal rights. All data were treated with strict confidentiality in line with institutional privacy policies, and the participants’ anonymity was fully protected.

### Data analysis

Statistical analyses were conducted using IBM SPSS (version 25.0) and AMOS (version 27.0). Continuous variables are presented as mean ± SD; categorical variables as frequencies and percentages. Normality was evaluated by skewness and kurtosis (acceptable if |skew| < 3 and |kurtosis| < 10). Missing values were handled by multiple imputation (m = 5), under the assumption that data were missing at random (MAR). Pearson correlations examined bivariate associations among the four constructs. Confirmatory factor analysis tested the measurement model prior to structural modelling. The hypothesized structural equation model was estimated using robust maximum likelihood estimation (MLR) in AMOS 27.0. Indirect effects were examined using bias-corrected bootstrapping with 5,000 resamples. Mediation was considered statistically significant if the 95% bias-corrected confidence interval did not include zero. Multi-group SEM compared pathways across educational levels (high: above high school vs. low: high school or below). Two-tailed tests were used; *p* < 0.05 was considered statistically significant, and *p* < 0.001 was highly significant.

## Results

### Participant characteristics

Table 1 summarizes demographic and clinical characteristics of the 321 patients with PNs. Participants had a mean age of 48.6 years (SD = 12.1; range: 18–80). Most were female (65.4%, *n* = 210), aged 40–59 years (52.0%), married (81.0%), urban residents (72.9%), and employed (70.4%). Ground-glass nodules were the most common type (59.5%). Slightly over half of the participants (51.1%) had multiple nodules, and the majority (83.8%) had a largest nodule size of 1–6 mm.

**Table 1:** Participant characteristics (*n* = 321).

Variable	Category	*n* (%)
Gender	Male	111 (34.6)
	Female	210 (65.4)
Age (years)	18–39	83 (25.9)
	40–59	167 (52.0)
	60–80	71 (22.1)
Marital status	Married	260 (81.0)
	Divorced/Widowed	31 (9.7)
	Single	30 (9.3)
Education level	Illiterate	11 (3.4)
	Primary school	35 (10.9)
	Junior high	62 (19.3)
	High school/Tech	51 (15.9)
	College	66 (20.6)
	Bachelor or above	96 (29.9)
Residence	Urban	234 (72.9)
	Rural	87 (27.1)
Employment	Employed	226 (70.4)
	Retired	63 (19.6)
	Unemployed	32 (10.0)
Monthly income (CNY)	<1,000	2 (0.6)
	1,001–3,000	50 (15.6)
	3,001–5,000	103 (32.1)
	5,001–10,000	86 (26.8)
	>10,000	80 (24.9)
Family history of lung cancer	Yes	44 (13.7)
	No	277 (86.3)
Smoking status	Current smoker	56 (17.4)
	Former smoker	26 (8.1)
	Never smoked	239 (74.5)
Nodule type	Ground-glass	191 (59.5)
	Solid	67 (20.9)
	Other	63 (19.6)
Number of nodules	Single	157 (48.9)
	Multiple	164 (51.1)
Nodule size (mm)	1–6 mm	269 (83.8)
	7–10 mm	48 (15.0)
	>10 mm	4 (1.2)
Disease duration	First diagnosis	165 (51.4)
	First revisit	102 (31.8)
	Multiple revisits	54 (16.8)

CNY, Chinese Yuan.

### Descriptive statistics and bivariate correlations

Table 2 presents the descriptive statistics and bivariate correlations of the study variables. The mean scores were 131.85 (SD = 34.96) for HISB, 79.54 (SD = 19.14) for health literacy, 40.81 (SD = 11.76) for illness perception, and 31.76 (SD = 8.31) for self-efficacy. All variables showed acceptable univariate normality (|skewness| < 3, |kurtosis| < 10). Health literacy showed positive correlations with both HISB (*r* = 0.464, *p* < 0.01) and self-efficacy (*r* = 0.453, *p* < 0.01), and a significant negative correlation with illness perception (*r* = −0.231, *p* < 0.01). Illness perception showed negative correlations with both HISB (*r* = −0.429, *p* < 0.01) and self-efficacy (*r* = −0.382, *p* < 0.01). Self-efficacy exhibited the strongest positive correlation with HISB (*r* = 0.497, *p* < 0.01), providing preliminary evidence for the hypothesized mediating pathways.

**Table 2:** Means, standard deviations, and variable correlations.

Variable	*M* ± SD	Skewness	Kurtosis	1	2	3	4
1. HISB	131.85 ± 34.96	0.09	−0.55	1			
2. HeLMS	79.54 ± 19.14	−0.25	−0.49	0.464^**^	1		
3. BIPQ	40.81 ± 11.76	0.04	−0.19	−0.429^**^	−0.231^**^	1	
4. GSES	31.76 ± 8.31	−0.06	−0.43	0.497^**^	0.453^**^	−0.382^**^	1

HISB, Health Information-Seeking Behavior; HeLMS, Health Literacy Management Scale; BIPQ, Brief Illness Perception Questionnaire; GSES, General Self-Efficacy Scale; M, mean; SD, standard deviation; Skewness, skewness; Kurtosis, kurtosis.

### Measurement model

Prior to testing the structural paths, a confirmatory factor analysis (CFA) was conducted for the four latent variables (health literacy, illness perception, self-efficacy, and Health Information-Seeking Behavior). Prior to testing the structural paths, a confirmatory factor analysis (CFA) established a well-fitting measurement model (*χ*^2^/df = 1.37, CFI = 0.994, TLI = 0.992, RMSEA = 0.034, SRMR = 0.028). The CFA results showed standardized factor loadings > 0.7, Average Variance Extracted (AVE) values > 0.5, and Composite Reliability (CR) values > 0.8, indicating strong convergent validity. Additionally, the square roots of the AVE values were greater than the absolute correlation coefficients between variables, demonstrating good discriminant validity.

### Structural equation model

**Figure 2:**
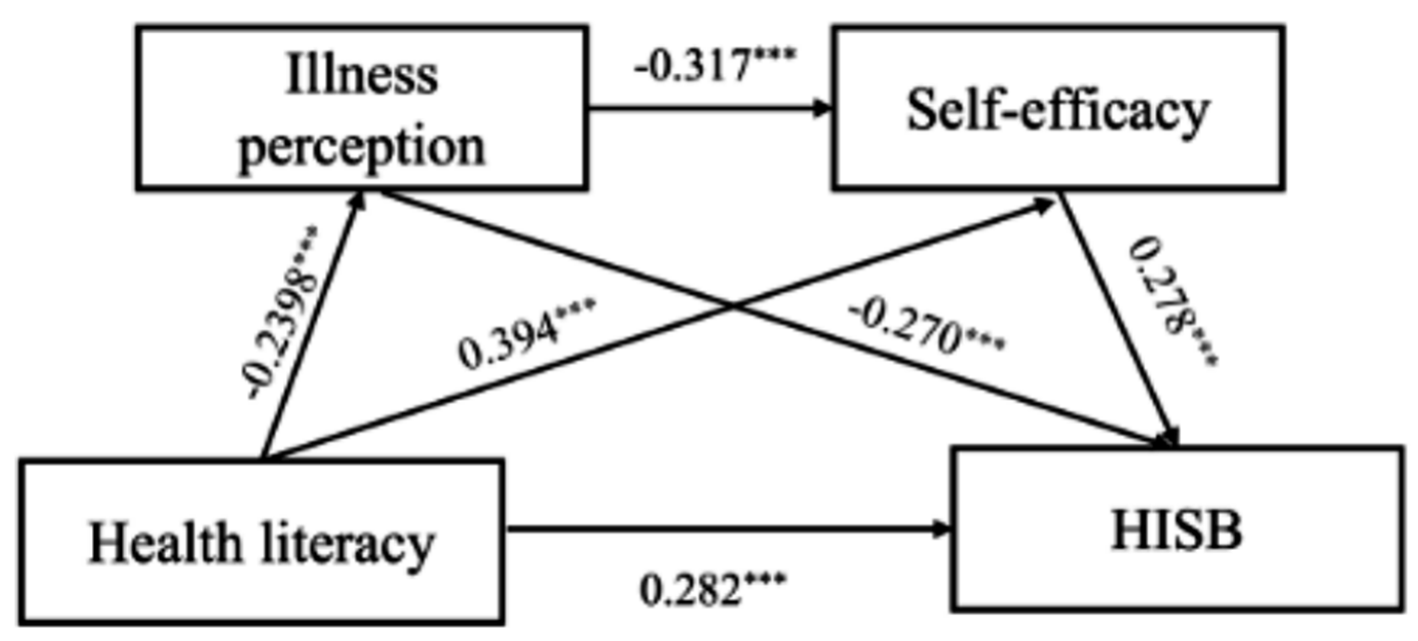
Path analysis of the relationships among health literacy, illness perception, self-efficacy, and Health Information-Seeking Behavior in patients with pulmonary nodules (*n* = 321).

The hypothesized IMB-based structural model demonstrated excellent overall fit to the data: *χ*^2^/df = 1.46, GFI = 0.905, RMSEA = 0.038, IFI = 0.982, CFI = 0.982, NFI = 0.946. Figure 2 illustrates the standardized path coefficients. Health literacy was significantly associated with HISB (*β* = 0.282, *p* < 0.001), illness perception (*β* = −0.239, *p* < 0.001), and self-efficacy (*β* = 0.394, *p* < 0.001). Illness perception was negatively associated with both HISB (*β* = −0.270, *p* < 0.001) and self-efficacy (*β* = −0.317, *p* < 0.001). Self-efficacy was positively associated with HISB (*β* = 0.278, *p* < 0.01). Together, these associations were consistent with the hypothesized IMB-based model specification.

### Direct, indirect, and total effects

As shown in Table 3, the total effect of health literacy on HISB was substantial and statistically significant [*β* = 0.477, 95% CI (0.381, 0.557), *p* < 0.001], indicating that health literacy was associated with HISB through both direct and indirect associations. The indirect effect through self-efficacy was the strongest [*β* = 0.110, 95% CI (0.062, 0.173), *p* < 0.001], followed by that through illness perception [*β* = 0.065, 95% CI (0.035, 0.108), *p* < 0.001]. Additionally, a statistically significant but modest serial mediation effect was observed [health literacy → illness perception → self-efficacy → HISB; *β* = 0.021, 95% CI (0.009, 0.040), *p* < 0.001], consistent with the hypothesized ordering.

**Table 3:** Direct, indirect, and total effects of variables in the structural model (*n* = 321).

Path	Estimate	SE	95% CI		*p*
			Lower	Upper	
Direct effects					
Health literacy → HISB	0.282	0.054	0.165	0.381	0.001
Health literacy → illness perception	−0.239	0.053	−0.343	−0.134	0.001
Health literacy → self-efficacy	0.394	0.057	0.284	0.502	0.001
Illness perception → HISB	−0.270	0.049	−0.366	−0.171	0.001
Self-efficacy → HISB	0.278	0.058	0.156	0.384	0.002
Illness perception → self-efficacy	−0.317	0.052	−0.415	−0.213	0.001
Indirect effects					
Health literacy → illness perception → HISB	0.065	0.018	0.035	0.108	0.001
Health literacy → self-efficacy → HISB	0.110	0.028	0.062	0.173	0.001
Health literacy → illness perception → self-efficacy → HISB	0.021	0.008	0.009	0.040	0.001
Total effect					
Health literacy → HISB	0.477	0.045	0.381	0.557	0.001

Estimate, standardized beta coefficient; SE, standard error; CI, confidence interval; *p*-values from bias-corrected bootstrapping; all paths remained statistically significant at *p* < 0.01.

### Multi-group path analysis by educational level

Measurement invariance was established across education groups (configural, metric, scalar, and strict invariance), with consistently excellent model fit (*χ*^2^/df = 1.40–1.43; RMSEA = 0.035–0.037; CFI, TLI, and IFI ≥ 0.964), supporting the validity of cross-group comparisons (see Supplementary Table 1). As shown in Table 4, all six hypothesized paths were statistically significant in both educational groups. The association between illness perception and self-efficacy was negative in both groups and was larger in magnitude in the high-education group (*β* = −0.377, *p* < 0.001) than in the low-education group (*β* = −0.244, *p* < 0.01). The association between health literacy and HISB was also stronger in magnitude in the low-education group (*β* = 0.302, *p* < 0.001) than in the high-education group (*β* = 0.253, *p* < 0.001). In addition, the association between self-efficacy and HISB was positive in both groups and was larger in the high-education group (*β* = 0.312, *p* < 0.001) than in the low-education group (*β* = 0.229, *p* < 0.05).

**Table 4:** Standardized path coefficients by educational level in multi-group structural equation modeling (*n* = 321).

	High education group		Low education group	
	Estimate	C.R.	Estimate	C.R.
Direct effects				
Health literacy→ illness perception	−0.230	−3.031^**^	−0.253	−2.728^**^
Health literacy→ self-efficacy	0.408	5.965^***^	0.373	4.161^***^
Illness perception → self-efficacy	−0.377	−5.277^***^	−0.244	−2.717^**^
Health literacy→ HISB	0.253	3.812^***^	0.302	3.514^***^
Illness perception→ HISB	−0.293	−4.255^***^	−0.234	−2.773^**^
Self-efficacy →HISB	0.312	4.043^***^	0.229	2.531^*^

Estimate, standardized beta coefficient; C.R., Critical Ratio; ^*^p < 0.05; ^**^p < 0.01; ^***^p < 0.001.

## Discussion

This study applied the IMB model to examine the associations between health literacy and HISB in patients with PNs, focusing on the roles of health literacy, illness perception, and self-efficacy. In a sample of 321 patients, the structural equation model demonstrated excellent fit, with all hypothesized associations statistically significant. A statistically significant but modest serial indirect effect (health literacy → illness perception → self-efficacy → HISB) was observed, which is consistent with the hypothesized ordering. The pattern of associations was broadly similar across educational levels, though path strengths varied, suggesting both universal and education-sensitive elements.

Health literacy was positively associated with HISB, both directly and indirectly through illness perception and self-efficacy. These findings align with existing evidence from chronic disease research, collectively reinforcing the critical role of health literacy as a foundational determinant of health-seeking behaviors (36, 37). Prior studies have consistently reported positive associations between higher health literacy and greater likelihood and quality of HISB, with some suggesting possible bidirectional links, namely that HISB may also enhance health literacy over time (38, 39). Importantly, in this study, health literacy was examined at the individual level. However, health literacy and HISB also unfold within broader healthcare contexts shaped by organizational demands, communication practices, and navigation complexity—factors that were not directly assessed here.

The mediation analysis found self-efficacy as the most influential mediator, accounting for 23% of the total effect of health literacy on HISB. This finding is consistent with the hypotheses of the IMB model, which posits behavioral skills as the proximal determinant of behavior (25). In contrast, illness perception accounted for 13.6% of the mediated effect. While consistent with Leventhal’s Common-Sense Model, illness perception can serve as motivational drivers of coping behavior (40). At the same time, illness perception is widely conceptualized as multi-dimensional, comprising cognitive representations, affective responses, and meaning-making processes; therefore, its influence on HISB may extend beyond a purely motivational role and may vary across time and context. This differential mediating capacity may be attributable to the inherent diagnostic ambiguity characterizing PNs. In the context of such uncertain health threats, cognitive appraisal alone may be insufficient to precipitate behavioral modification, and affective responses and system-level cues may also shape information seeking.

The serial mediation analysis revealed a pattern of associations linking these variables, conceptually consistent with the IMB framework’s interplay of informational, motivational, and skill-based components (25). While previous research has established a direct association between health literacy and HISB (23), our analysis provides support for a theoretically specified pathway linking health literacy to HISB via illness perception and self-efficacy. Although the proposed sequential mediation is theoretically grounded, the cross-sectional design precludes causal inference, and the observed pathway should be interpreted as a pattern of associations rather than a definitive temporal sequence. Conceptual overlap between illness perception and self-efficacy may also influence the observed relationships.

Multi-group analysis confirmed the stability of the SEM across patients with different educational backgrounds. Noteworthy, threatening illness-perception had negative effect on self-efficacy, which was more pronounced among highly educated patients. From the perspective of information processing theory, this difference may originate from distinct cognitive pathways (41). Highly educated patients tend to engage in central-route processing by logical thinking. Consequently, if they develop a catastrophic illness representation, it may lead to a more marked decline in self-efficacy (42). In contrast, patients with lower education levels primarily rely on peripheral-route processing largely by intuition: their judgments are more influenced by the opinion of physicians, even non-professional folks (43). These findings suggest that clinicians should adopt stratified strategies based on patients’ educational levels and corresponding information-processing styles.

While the IMB model provides a useful heuristic for organizing individual-level behavioral determinants, it does not fully capture the broader cognitive, affective, social, and system-level influences known to shape HISB. In addition, the present operationalization of health literacy emphasizes personal capability; yet HISB is also contingent on relational and organizational health literacy (e.g., how information is communicated, whether services are navigable, and whether patients experience the system as accessible and acceptable). These unmeasured determinants may interact with individual health literacy and self-efficacy, shaping whether information seeking is initiated, sustained, and translated into appropriate care decisions. More recent integrative behavioral frameworks, such as the Integrated Screening Action Model (I-SAM) (44) and the Determinants of Screening Uptake Model (DOST) (45), address these limitations by simultaneously considering cognitive, affective, social, and health-system determinants, and explicitly incorporating health literacy to explain screening-related care seeking within multilevel contexts. Accordingly, the present findings should be interpreted as delineating specific individual-level psychological pathways that likely operate within broader multilevel behavioral systems, rather than as a substitute for more comprehensive models. In this sense, the IMB-based model here captures partial behavioral pathways and can complement—rather than replace—integrative screening frameworks.

### Implications for clinical practice

Although this study focused on individual-level psychological pathways, HISB during lung nodule surveillance and lung cancer screening is embedded in multilevel systems that include clinical communication and service structures. In line with integrative frameworks such as I-SAM and DOST (44, 45), the findings may inform several practice-oriented strategies. First, drawing specifically on the DOST framework’s emphasis on literacy (45), tailored, literacy-sensitive educational materials may be relevant, with in-depth interpretations of authoritative guidelines for highly educated patients and accessible content disseminated through social media and other platforms for those with lower education. Second, strategies addressing illness-related anxiety through nurse-coordinated reframing support may be considered. Third, fostering a clinical environment that encourages questions could be valuable. Equipping patients with practical skills, such as using question-prompt lists, identifying credible resources, and applying specific communication techniques, might be considered. Follow-up clinics or digital platforms can be used to deliver this support, with the aim of enhancing patients’ self-efficacy. At the same time, improving HISB and screening engagement likely requires concurrent attention to system-level determinants—such as clarity and consistency of provider communication, availability of trustworthy information channels, appointment access, and service organization—so that individual capability is matched by an enabling care environment. While acknowledging the cross-sectional nature of the data, these results suggest avenues for literacy-sensitive, stratified approaches in lung nodule surveillance and lung cancer screening programs to address health literacy gaps and reduce inequities.

### Strengths, limitations, and future directions

Strengths include the integration of health literacy, illness perception, and self-efficacy within an IMB-based model of HISB, rigorous SEM and multi-group analysis, and attention to education-related variations. However, several limitations should be acknowledged. First, the cross-sectional design restricts causal inference. Second, self-report measures are subjective, which may yield social desirability and common-method bias. Third, although validated instruments were employed, the overall length of the questionnaire was relatively extensive, which may have increased response burden and fatigue among patients and potentially influenced response quality. Fourth, participants were recruited from two tertiary hospitals in a single city, which may limit the generalizability of the findings to broader populations. Finally, this study did not assess system-level determinants that may shape HISB and interact with individual psychological factors. Future research should employ longitudinal, multi-center designs with diverse samples, objective measures where feasible, and multilevel models that explicitly incorporate provider communication, organizational practices, health-system navigation demands, and integrative health behavior frameworks to more comprehensively explain HISB and inform scalable interventions.

## Conclusion

In conclusion, health literacy, illness perception, and self-efficacy were associated with HISB among patients with PNs. The observed pattern of indirect associations, including a modest serial indirect effect, is consistent with the hypothesized ordering within the IMB framework. From a practical perspective, the results highlight psychosocial factors that may inform the design of supportive, literacy-sensitive strategies. Healthcare professionals may tailor education and communication to patients’ health literacy levels and informational needs, incorporate skill-building approaches to support self-efficacy, and create environments that encourage questions and facilitate access to credible information to support informed shared decision-making and patient engagement during surveillance.

## Data Availability

The original contributions presented in the study are included in the article/Supplementary material, further inquiries can be directed to the corresponding authors.
