# Supplementary material for: The association between health literacy and Health Information-Seeking Behavior among pulmonary nodule patients: a serial mediation of illness perception and self-efficacy
Source: Front Public Health. 2026 Mar 18;14:1773518. doi: 10.3389/fpubh.2026.1773518 (PMC13038900; doi:10.3389/fpubh.2026.1773518)
Supplement: Supplementary file 1 [file Table_1.docx]

**Table S1**  Model Fit Indices for Multi-Group Structural Equation Modeling

| **Model** | **CMIN/DF** | **NFI** | **RFI** | **IFI** | **TLI** | **CFI** | **RMSEA** |
| --- | --- | --- | --- | --- | --- | --- | --- |
| Unconstrained | 1.423 | 0.901 | 0.89 | 0.968 | 0.965 | 0.968 | 0.036 |
| Measurement weights | 1.406 | 0.898 | 0.891 | 0.968 | 0.966 | 0.968 | 0.036 |
| Structural covariances | 1.4 | 0.898 | 0.892 | 0.968 | 0.966 | 0.968 | 0.035 |
| Measurement residuals | 1.425 | 0.891 | 0.89 | 0.965 | 0.964 | 0.965 | 0.037 |
